# The complete mitochondrial genome of *Agelasta perplexa* Pascoe (Coleoptera: Cerambycidae)

**DOI:** 10.1080/23802359.2021.1915712

**Published:** 2021-05-19

**Authors:** Yanting Li, Runkai Chen, Hanzhu Jia, Xuemin Lei, Jiayi Ma, Songqing Wu

**Affiliations:** a College of Forestry, Fujian Agriculture and Forestry University, Fuzhou, China; b Key Laboratory of Integrated Pest Management in Ecological Forests, Fujian Province University, Fujian Agriculture and Forestry University, Fuzhou, China

**Keywords:** mtDNA, phylogenetic analysis, *Agelasta perplexa*

## Abstract

*Agelasta perplexa* Pascoe is a mulberry borer that threatens the health of the plant. This study revealed the length of the complete mitochondrial genome of *A. perplexa* which consists of 15,552 bp length with 39.8% A, 12.8% C, 8.4% G, and 39.1% T, respectively. The GC content of whole mitochondrial genome is 21.1%. The complete mitochondrial genome encodes 12 protein-coding genes (PCGs), 22 tRNAs, two rRNAs, and one AT-rich region. This study can facilitate further research about genetic evolution as well as prevention and control strategy of *A. perplexa*.

*Agelasta perplexa* Pascoe (Coleoptera: Cerambycidae) is mainly distributed in Southeast China. The dark body is 11–18 mm long with intensive reddish-brown and gray-white spots. *A. perplexa* is predominant pest of *Morus alba* L., causing extensive damage to the plant (Yamasako and Ohbayashi 2012), so it is particularly important to control it. However, species information of *A. perplexa* is not enough. In order to better know and reveal the genetic and evolutionary information of *A. perplexa*, so as to better carry out the prevention and treatment work, the complete mitochondrial genome of *A. perplexa* was determined in this study.

There were five adults of *A. perplexa* collected from Lianjiang, Fujian province, China (26.32836 N, 119.80018 E) by the traps with sex pheromone. The specimens were preserved at −80 °C in the Key Laboratory of Integrated Pest Management in Ecological Forests, Fujian Agriculture and Forestry University (Songqing Wu, dabinyang@126.com) under the voucher number TN-202010. To construct the sequencing library and obtain sequence information from mitochondrial DNA, the TruSeq DNA Sample Preparation Kit (Vanzyme, Nanjing, China) was utilized to extract the total DNA and the samples were purified by the QIAquick Gel Extraction Kit (Qiagen GmbH, Hilden, Germany). Then, the DNA library was contrusted using transposase method. Agencourt Spriselect was used to purify the library and perform size selection, and the library with a fragment peak value of 300 bp was selected for sequencing. Furthermore, the mitochondrial genome was 2 × 150 paired-end sequenced by the Illumina Hiseq 2500 (Illumina, San Diego, CA) at Genesky Biotechnologies Inc. (Shanghai, China). After quality control and filtration, the total 58,116,830 clean reads were obtained from 60,728,446 raw reads. Then, MitoZ and metaSPAdes were applied to assemble the clean reads (Nurk et al. 2017). GeSeq was used to annotate the mitogenome of samples followed by individually correction in Geneious using a reference genome (GenBank accession no. KY292221) (Masters et al. 2015; Michael et al. 2017).

The results of assembly and annotation indicated that length of the complete mitochondrial genome of *A. perplexa* is 15,552 bp. The GC content of whole mitochondrial genome is 21.1%. The complete mitochondrial genome encodes 12 protein-coding genes (PCGs), 22 tRNAs, two rRNAs, and a 905 bp AT-rich region. Twelve PCGs are 10,710 bp in total, encoding 3570 amino acids. Six PCGs (COII, COIII, ND3, ND4, ND4L, CYTB) start with a typical ATN codon, while the other PCGs codons are unusual; nine PCGs (ND2, COI, COII, ATP6, COIII, ND5, ND4, ND4L, ND6) stop with codon TAA, and three PCGs (ND3, CYTB, ND1) stop with codon TAG. The rrnS and rrnL genes are 781 bp and 1275 bp in length, respectively.

In order to gain insight into the phylogenetic relationships of *A. perplexa*, MAFFT 7.0 (Kazutaka and Standley 2013) was used for sequence alignment according to the complete mitochondrial genome sequence of *A. perplexa*. Then using a Lepidoptera species *Cnidocampa flavescens* Walker (GenBank accession no. KY628213) as an out-group, a maximum-likelihood phylogenetic tree with 1000 bootstraps was constructed using MEGA 7.0 to reconstruct the phylogenetic position of *A. perplexa* with 16 related Coleoptera species (Kumar et al. 2016). The resultant ML trees showed that the *A. perplexa* was clustered together with *Agapanthia daurica* Ganglbauer, and constituted a monophyletic group with 10 species belonging to the subfamily Lamiinae. The monophyletic Lamiinae species was assigned to the sister group to the clade of Orsodacnidae and Cerambycidae that consists of Orsodacne, Aseminae, Lepturinae, and Spondylinae in this study (Figure 1). The results consolidate the existing knowledge of *A. perplexa.* The availability of the complete mitochondrial genome of *A. perplexa* allows further research about the genetic evolution and control tactics of *A. perplexa* can be facilitated.

**Figure 1.**
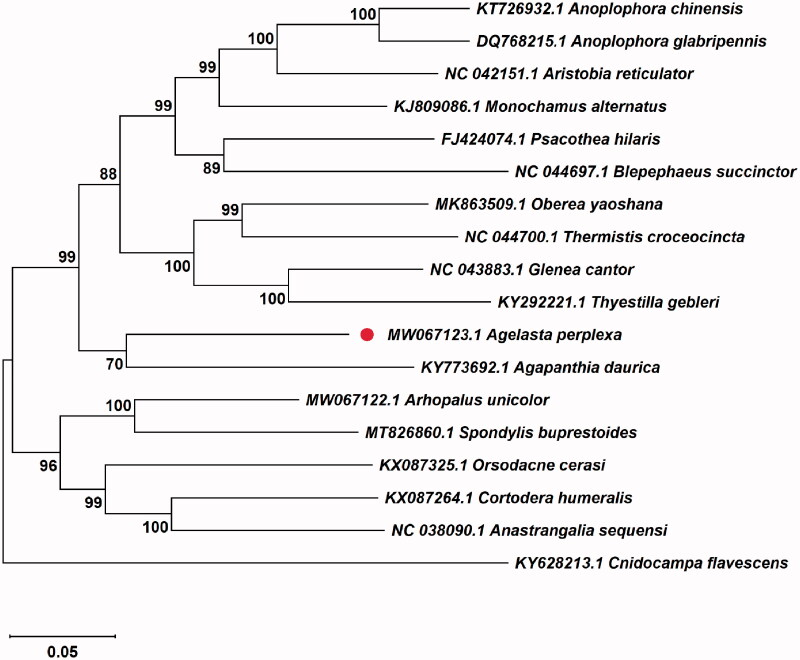
Maximum-likelihood tree of *A. perplexa*, *C. flavescens*, and 16 species from Coleoptera according to the genome sequence alignment. Numbers labeled on the branch represent bootstrap values.

## Data Availability

Mitogenome data supporting this study are openly available in GenBank at nucleotide database, https://www.ncbi.nlm.nih.gov/nuccore/MW067123, Associated BioProject, https://www.ncbi.nlm.nih.gov/bioproject/PRJNA681753, BioSample accession number at https://www.ncbi.nlm.nih.gov/biosample/SAMN16967690, and Sequence Read Archive at https://www.ncbi.nlm.nih.gov/sra/SRR13172076.
